# Chemopreventive effect of modified zeng-sheng-ping on oral squamous cell carcinoma by regulating tumor associated macrophages through targeting tnf alpha induced protein 6

**DOI:** 10.1186/s12906-024-04593-0

**Published:** 2024-07-27

**Authors:** Jiaqi Wang, Feiran Lin, Yongxiang Zhou, Yuyi Cong, Sen Yang, Sujuan Wang, Xiaobing Guan

**Affiliations:** 1 Department of Oral Medicine, Beijing Stomatological Hospital, Capital Medical University, Beijing, 100050 China; 2 State Key Laboratory of Bioactive Substance and Function of Natural Medicines, Institute of Materia Medica, Chinese Academy of Medical Sciences and Peking Union Medical College, Beijing, 100050 China

**Keywords:** Traditional chinese medicine, Chemoprevention, Oral squamous cell carcinoma, Tumor associated macrophages

## Abstract

**Background:**

Oral squamous cell carcinoma (OSCC) is the most common malignancy of the head and neck. Zeng-Sheng-Ping, composed of *Sophora tonkinensis* Gagnep., *Bistorta officinalis* Delarbre, *Sonchus arvensis* L., *Prunella vulgaris* L., *Dioscorea bulbifera* L., and *Dictamnus dasycarpus* Turcz., was regarded as an anti-cancer drug with significant clinical efficacy, but was discontinued due to liver toxicity. Our research group developed a modified Zeng-Sheng-Ping (ZSP-M) based on original Zeng-Sheng-Ping that exhibited high efficiency and low toxicity in preliminary investigations, although its pharmacodynamic mechanism is still unclear. Here, we aimed to elucidate the pharmacodynamic material basis of ZSP-M and investigate its chemopreventive effect on OSCC by modulating tumor associated macrophages (TAMs).

**Methods:**

Components of ZSP-M were characterized using ultra-performance liquid chromatography-mass spectrometry. Chemopreventive effect induced by ZSP-M against experimental oral cancer was investigated using the 4-nitroquinoline N-oxide precancerous lesion mouse model. RNA sequencing analysis was used to gain a global transcriptional view of the effect of ZSP-M treatment. A cell co-culture model was used to study the targeted effect of ZSP-M on TAMs and the biological properties of OSCC cells and to detect changes in TAM phenotypes. The binding of ZSP-M active compounds to TNF alpha induced protein 6 (TNFAIP6) protein was analyzed by molecular docking and dynamic simulation.

**Results:**

Forty main components of ZSP-M were identified, the most abundant of which were flavonoids. ZSP-M inhibited the degree of epithelial dysplasia in precancerous lesions by inhibiting the expression of the TNFAIP6 and CD163 proteins in the precancerous lesions of the tongue. ZSP-M inhibited proliferation, colony formation, migration and invasion of SCC7 cells by targeting TAMs. ZSP-M reduced the expression of CD163^+^ cells, inhibited the expression of TNFAIP6 protein, *Arg1* mRNA and *Il10* mRNA in TAMs, and reduced IL-10 cytokine release in the co-culture environment. This effect was maintained after the addition of recombinant TNFAIP6 protein. Computer simulations showed that trifolirhizin and maackiain are well-connected to TNFAIP6.

**Conclusions:**

ZSP-M counteracts the immunosuppressive action of TAMs by specific targeting of TNFAIP6, thereby exerting chemopreventive activity of OSCC.

**Supplementary Information:**

The online version contains supplementary material available at 10.1186/s12906-024-04593-0.

## Background

Oral squamous cell carcinoma (OSCC) is the most common type of oral cancer, with a high incidence in Southern Asia [1]. In most patients, OSCC develops from oral potentially malignant disorders (OPMDs). Oral leukoplakia is a common type of OPMD with a malignancy rate ranging from 0.13% to 34% [2]. When a lesion develops into OSCC, patients require surgery, radiotherapy, chemotherapy, and even targeted therapy, which may heavily reduce their quality of life [3]. Moreover, the current prognosis of OSCC remains unsatisfactory. The 5-year survival rate for advanced OSCC is < 20%; however, timely intervention has the potential to increase this to 80% [4].

The use of natural or synthetic chemopreventive drugs to prevent carcinogenesis has been an important focus of OSCC disease management for decades, although the effects of some drugs have been overestimated [5, 6]. Clinical experience have demonstrated that Zeng-Sheng-Ping (ZSP, designated original ZSP/ZSP-O, also referred to as antitumor B), comprising of *Sophora tonkinensis* Gagnep. [*Fabaceae*; Sophorae tonkinensis radix et rhizome, ST, 山豆根], *Bistorta officinalis* Delarbre. [*Polygonaceae*; Bistortae rhizome, BO, 拳参], *Sonchus arvensis* L. [*Asteraceae*; Sonchi arvensis herba, SA, 北败酱], *Prunella vulgaris* L. [*Lamiaceae*; Prunellae spica, PV, 夏枯草], *Dioscorea bulbifera* L. [*Dioscoreaceae*; Dioscoreae bulbiferae rhizome, DB, 黄药子], and *Dictamnus dasycarpus* Turcz. [*Rutaceae*; Dictamni Cortex, DD, 白鲜皮], is one of the few medicines that block the malignant transformation of precancerous lesions in the digestive tract. Our research group has reported that ZSP-O has an excellent therapeutic effect on OPMD such as leukoplakia and lichen planus [7, 8]. Unfortunately, the clinical application of ZSP-O has been limited by the incidence of sporadic liver injury cases [9]. For more than 30 years, our research group has focused on improving ZSP-O and we have attempted to alter the extraction methods. Moreover, we have adopted a topical drug-delivery approach to reduce the metabolic toxicity of oral administration [10]. An NMR-based metabolomic approach combined with LC–MS profiles obtained in a recent study implicated diosbulbin B, obacunone, and fraxinellone as possible toxic components of ZSP-O [11]. By removing the hepatotoxic constituents from ZSP-O, we developed a modified ZSP (ZSP-M) [12] with low hepatotoxicity.

In our previous in vitro study, it was confirmed that ZSP-M and ZSP-O have almost an equal ability to inhibit the biological characteristics of OSCC cells, including proliferation, clonogenesis, invasion, migration and apoptosis [13]. Further experiments on cheek pouch cancer and chronic toxicity in the experimental golden hamster model showed that ZSP-M exhibited superior efficacy compared to ZSP-O. Notably, ZSP-M has demonstrated a significant reduction in hepatotoxicity in animal studies, making it a safer alternative to ZSP-O [14].

In order to clarify the specific mechanism underlying the anticancer activity of ZSP-M, we employed the classical 4-Nitroquinoline N-oxide (4NQO) precancerous lesion mouse model in this study. The morphological and histological pattern of mouse tongue induced by 4NQO is similar to oral leukoplakia observed in humans, and simulates OPMD, thus representing a suitable model for studying the ability of ZSP-M to play the role of cancer chemoprevention [15]. Additionally, the mouse is a highly suitable species for sequencing analysis and mechanistic studies.

Recently, a series of studies demonstrated that the level of tumor associated macrophages (TAMs) is closely related to the progression and poor prognosis of epithelial cell-derived tumors such as oral [16], esophageal [17], and gastric cancers [18]. TAMs also participate in the transformation of leukoplakia into oral cancer [19, 20]. TCMs such as ginseng, astragalus [21], panax notoginseng [22], and modified Si-Jun-Zi decoction [23] have been shown to suppress the growth of carcinomas and prevent recurrence or metastasis by regulating TAMs. In our previous network pharmacology and in vitro studies [13], we demonstrated that the constituents of ZSP-M exert regulatory effects on differentially expressed genes (DEGs) associated with OSCC, which are enriched in pathways related to tumor progression and immune response. These findings prompted us to further investigate the regulatory effect of ZSP-M on TAMs.

In this study, we aimed to elucidate the pharmacodynamic material basis of ZSP-M and investigate the mechanism of its chemopreventive effect on OSCC by modulating TAMs through a combination of in vitro and in vivo experiments combined with computer simulations.

## Materials and methods

### Preparation of ZSP-O and ZSP-M

The herbs used to prepare ZSP-O and ZSP-M were purchased from the Bai-Ta-Si TCM store (Beijing, China), and authenticated based on Chinese Pharmacopoeia. The recipe formula was as follows: ST 420 g, BO 420 g, SA 420 g, PV 420 g, DB 100 g, and DD 210 g [24]. To prepare ZSP-O, all botanical drugs were boiled in 19.9 L water for 2 h (× 2). After filtration, the water solution was concentrated to obtain ZSP-O extract. To prepare ZSP-M, DB and DD were boiled in 3.1 L water for 2 h (× 2). After concentrated to approximately 300 mL, the aqueous solution was partitioned with ethyl acetate (× 3) to remove the hepatotoxic constituents. The obtained aqueous phase was then mixed with the water extract of the other four botanical drugs [boiled in 16.8 L water for 2 h (× 2)], and then concentrated to obtain ZSP-M extract [11]. All ZSP water solutions were concentrated to solid extract and stored in refrigerator at 4℃ before usage.

### UPLC-MS analysis

ZSP-O and ZSP-M were weighed (52 mg) and sonicated in 1040 μL ddH2O (containing 4 μg/mL L-2-chlorophenylalanine) in an ice bath for 60 min. The solution was filtered using a 0.22 μm micropore membrane and 5 μL was collected for analysis. UPLC-MS analysis was performed using a system consisting of ACQUITY UPLC I-Class HF ultra-high performance liquid chromatography in series with a QE high-resolution mass spectrometer. Chromatographic separation was performed using an ACQUITY UPLC HSS T3 (100 mm × 2.1 mm, 1.8 μm) column (Waters, USA) at column temperature of 45 ℃. The mobile phase consisted of 0.1% formic acid solution (A) and acetonitrile (B) with a flow rate of 0.35 mL/min, a injection volume of 5 μL and the PDA scanning range of 210–400 nm. A linear gradient of 5% B (4 min) → 30% B (4 min) → 50% B (2 min) → 80% B (4 min) → 100% B (1 min) was used for elution. The samples were analyzed using the Thermo Obitrap QE mass spectrometer with HESI ion source. The analysis was performed using both positive and negative ion scanning modes, employing the Full MS/dd-MS2 scanning mode and a data dependent acquisition data collection mode. The Full ms resolution was 70,000, and the MS/MS resolution was 17,500.

The original data were processed by Progression QI v3.0 software (Nonlinear Dynamics, Newcastle, UK) for baseline filtering, peak recognition, integration, retention time correction, peak alignment, and normalization. Set the total relative peak area of components to 100% to obtain a qualitative and quantitative result data matrix. The identification of compounds was based on mass numbers, fragments ion, and isotope distribution. A TCM database developed by Oebiotech Co. Ltd. was used to clarify the composition. This database is a plant sample database that contains detailed information on over 5000 standard ingredients of TCM. It includes fundamental details such as classification, mass number, fragment ion, and retention time, along with crucial biological data like the source and component classification.

### In vivo experimental verification

#### 4NQO precancerous lesion mouse model

A precancerous lesion model was established in mice according to the animal protocol approved by the Animal Ethical and Welfare Committee of the Beijing Stomatological Hospital (ethics approval number: KQYY-202104–001). All methods were performed in accordance with the ARRIVE guidelines [25]. Male C57BL/6 mice (aged 6 weeks, weighing 20 ± 2 g, *n* = 18) were purchased from the Sibeifu Company (Beijing, China). Mice were allocated to four groups. The control group (*n* = 3) was fed a normal diet. According to the ARRIVE guidelines, it is recommended to minimize the number of animals used in experiments to a statistically valid extent. Our preliminary experiments conducted in the early stage demonstrated that employing a sample size of 5 animals per group in the experimental groups yielded stable and statistically significant results. Experimental groups were treated with 4NQO (Sigma, N8141) dissolved in drinking water (100 mg/L for 12 weeks. At the beginning of the 13th week, mice in the experimental groups were randomly divided into three groups using a Microsoft Excel-based random order generator. In the 4NQO model group (*n* = 5), water containing 4NQO was replaced with regular tap water, while water was replaced with water containing ZSP-O and ZSP-M (4.341 g/L) in the ZSP-O (*n* = 5) and ZSP-M (*n* = 5) groups, respectively. The administration dosage was calculated using the following formula:

1 $$Dosage\,per\,mouse = 80 mg/kg (the\,clinical\,dose\,used\,in\,humans) \times 12.3 (the\,conversion\,factor\,in\,mouse)$$

To ensure the daily intake of drugs and reduce damage to the esophageal mucosa by intragastric administration, ZSP-O/M was added to drinking water, which was changed every 3 days.

Mice were anesthetized and unconscious with pentobarbital sodium (50 mg/kg) and sacrificed with cervical dislocation at the end of the 22nd week, and their tongues were removed and imaged. Hematoxylin and eosin (HE) staining was utilized to assess epithelial dysplasia in accordance with the World Health Organization Classification of Head and Neck Tumors, 5th Edition [26]. Immunohistochemical staining was performed to determine the expression levels of TNFAIP6 (sc-65886, Santa Cruz, 1:50) and CD163 (A8383, Abclonal, 1:100) proteins. TNFAIP6 expression was assessed through a comprehensive evaluation of both staining intensity and area, while CD163 expression was quantified based on the positive cell number. The researchers utilized the image analysis software, cellSens Standard, to analyze these results.

#### RNA sequencing analysis

The middle (third) part of the tongue (site without evident exophytic tumor) in the control group, 4NQO model group and ZSP-M group was used for RNA sequencing (RNA-Seq) analysis (3 mice randomly selected from each group). The RNA-Seq analysis was performed by Oebiotech Co. Ltd. (Shanghai, China) using mRNA Seq Sample Prep kits (Illumina). A corrected *P*-value of 0.05 and log2 (fold change) of 1 were set as the threshold for significant DEGs.

### In vitro experimental validation

#### Cell lines and culture

RAW264.7 cells (murine macrophage cells) and the SCC7 cells (murine squamous cell carcinoma cells) were purchased from the American Type Culture Collection (ATCC; Manassas, VA, USA). The cells were cultured in Dulbecco’s modified Eagle’s medium (DMEM; Gibco) supplemented with 10% fetal bovine serum (FBS; Gibco), 2 mM L-glutamine (Gibco), 100 U/mL penicillin and 100 µg/mL streptomycin (Gibco) and incubated at 37 °C in a humidified atmosphere under 5% CO_2_. Cells were passaged when they reached 70%–80% confluence.

#### Cell co-culture

RAW264.7 cell conditioned medium (CM) was employed to assess the proliferation, migration, and invasion of SCC7 cells within the co-culture setting. RAW264.7 cells were treated for 24 h with ZSP-M at concentrations of 0, 5, and 20 μg/mL. The cells were then seeded in culture plates in DMEM supplemented with 0.2% FBS. After incubation for a further 24 h, the medium was collected, subjected to bacterial filtration, and supplemented with 10% FBS, 2 mM L-glutamine, 100 U/mL penicillin and 100 µg/ml streptomycin to prepare the CM.

Phagocytosis by TAMs was assessed through direct contact co-culture. The phenotype of TAMs was evaluated using a Transwell (0.4 μm) co-culture system containing SCC7 cells and RAW264.7 cells in the upper and lower compartments, respectively, at a 1:1 ratio.

#### Effect on OSCC cells

Colony formation assays were used to determine the proliferative capacity and population characteristics of cells. Cells were seeded in 6-well plates (400 cells/well) and cultured for 14 days. After staining with crystal violet, clusters containing > 50 cells were considered one colony. The number of colonies was manually counted using a microscope.

Scratch assays were used to determine OSCC cell migration. SCC7 cells were seeded in 6-well plates and cultured to 90% confluence before a consistent scratch was created in the cell monolayer. Images were captured at the same location on days 0, 1, and 2 in order to compare the extent of cell migration.

OSCC cell invasiveness was assessed using Transwells (8 μm, Corning, USA) coated with Matrigel (Corning, BioCoat, 356,234). Cells were seeded in the upper chamber containing CM (supplemented with 0.2% BSA without FBS), while the lower chamber contained DMEM supplemented with 10% FBS. The cells were then incubated for 2 days, before the cells that traversed the Matrigel from the upper to the lower chamber were fixed, stained, and counted.

#### Effect on TAMs

SCC7 cells were pre-labeled with carboxyfluorescein succinimidyl ester and seeded in 6-well plates (100,000 cells/well). RAW264.7 cells were pre-treated for 24h with various concentrations of ZSP-M before co-culture with SCC7 cells (300,000 cells/well). After incubation for 2 h in the absence of light, cells were harvested and labeled with F4/80 antibody (Biolegend) to specifically target macrophages, which were detected by flow cytometry. The proportion of macrophages engaged in phagocytosis of tumor cells was calculated according to the following formula:

$$\text{Phagocytosis rate }= \text{number of double positive cells}\times100\%/\text{number of F4}/80^+\text{cells.}$$

RAW264.7 cells were pre-treated for 24 h with various concentrations of ZSP-M before co-culture with SCC7 cells in Transwell chambers separated by a membrane (pore size 0.4 μm). After incubation for a further 24 h, RAW264.7 cells in the lower chamber were harvested and incubated for 1 h with CD163 antibody. The proportion of CD163^+^ cells was then assessed using flow cytometry.

TNFAIP6 protein expression was analyzed by Western blotting. Total proteins were extracted from cells using RIPA buffer (Beyotime BioTech, Shanghai, China) and concentration was determined using a Bradford protein assay kit (KeyGen BioTech). Total proteins were separated by 4%–12% gradient polyacrylamide gel electrophoresis and transferred to a polyvinylidene fluoride membranes (BioRad) using a Trans-blot Turbo. The membrane was incubated with 5% non-fat milk solution to block non-specific binding before incubation overnight with anti-TNFAIP6 (A6419, Abclonal, 1:1,000) and anti-β-tubulin (AC021, Abclonal, 1:3,000) primary antibodies. Subsequently, the membrane was incubated with the corresponding secondary antibody (anti-IgG, 1:2,000) for 1 h at room temperature. Bands were visualized using Clarity™ Western ECL Substrate (BioRad) and detected with a BioRad imaging system.

*Arg1* and *Il10* mRNA levels in RAW264.7 cells were quantified by RT-qPCR*.* Total RNA was isolated using TRIzol reagent (Sigma-Aldrich, USA) and was reverse transcribed into cDNA with PrimeScript™ RT reagent kit (TaKaRa, Japan). cDNA was amplified in duplicate using a thermal cycler (9700 HT RT-PCR, Applied Biosystem, UK) with TB Green™ Premix ExTaq™ II (TakaRa). The following primers were used: *Arg1* Forward Primer 5'-CATATCTGCCAAAGACATCGTG-3', Reverse Primer 5'-GACATCAAAGCTCAGGTGAATC-3'. *Il10* Forward Primer 5'-TTCTTTCAAACAAAGGACCAGC-3', Reverse Primer 5'-GCAACCCAAGTAACCCTTAAAG-3'. Mouse GAPDH was used as the endogenous reference (B661304) and all primers were purchased from Sangon Biotech.

IL-10 levels in RAW264.7 culture medium were determined using the Mouse IL-10 ELISA kit (RK00016, Abclonal) according to the manufacturer’s instructions.

To investigate the role of TNFAIP6 in the mechanism by which ZSP-M targets TAMs to inhibit OSCCs, we conducted a rescue experiment by adding recombinant TNFAIP6 protein (100 ng/mL) to the co-culture environment.

### Molecular docking and molecular dynamics simulation

Molecular docking was used to investigate the theoretical interaction between the active compounds in ZSP-M and target protein TNFAIP6. The crystal structure of TNFAIP6 protein was obtained from the Uniprot database, the 3D structures of compounds (Trifolirhizin, Maackiain, Matrine, Oxymatrine, Chlorogenic acid, Puerarin, Rutin, Chicoric-acid, Oxysophoridine, Miquelianin, Vitexin-2-O-rhamnoside, and Caffeic acid) were obtained from the Pubchem database. Energy minimization was performed under the MMFF94 force field. After processing the receptor protein using PyMol 2.5.1, including removing waters, salt ions, and small molecules, semi-flexible molecular docking simulation was performed using AutoDock Vina 1.1.2 software. The docking cube size was set to 65 × 65 × 65 Å^3^ to wrap around the entire protein structure. In addition, ADFR suite 1.0 was used to convert all processed molecules and receptor proteins into the PDBQT format. When docking, the exhaustiveness was set to 32, while the remaining parameters remain at their default settings. The conformation with the highest score was considered as the bound conformation. PyMol 2.5.1 was utilized for visual analysis of the results.

Molecular dynamics simulation was used to further investigate the binding energy and stability of the interaction. Initial structures for isothermal and isobaric phylogenetic simulations in AMBER 18 software were generated by docking trifolirhizin or maackiain with TNFAIP6 complexes. The molecular mechanics/generalized born surface area method [27] was employed to calculate the binding energy through a 50-ns simulation trajectory using the following formula:

2 $$\Delta\text{G}_\text{bind}=\Delta\text{G}_\text{complex}-(\Delta\text{G}_\text{receptor}+\Delta\text{G}_\text{ligand})=\Delta\text{E}_\text{internal}+\Delta\text{E}_\text{VDW}+\Delta\text{E}_\text{elec}+\Delta\text{G}_\text{GB}+\Delta\text{G}_\text{SA}$$

The ΔE_internal_, ΔE_VDW_, ΔE_elec_, ΔG_GB_ and ΔG_SA_ represent the internal energy, Van der Waals interaction, electrostatic interaction, polar solvation free energy, and nonpolar solvation free energy, respectively. Binding stability was assessed on the basis of root mean square deviation (RMSD) and root mean square fluctuation (RMSF).

### Statistical analysis

Statistical analysis was performed using SPSS version 17.0 for windows (SPSS Inc. Chicago, IL, USA). Data were expressed as the mean ± standard error (SE) and analyzed by the Shapiro–Wilk normality test and Levene’s test. Differences between groups were evaluated using a t-test or one-way analysis of variance followed by Tukey's HSD test. *P* < 0.05 was considered to indicate statistical significance.

## Results

### UPLC-MS analysis of ZSP-M

**Fig. 1:**
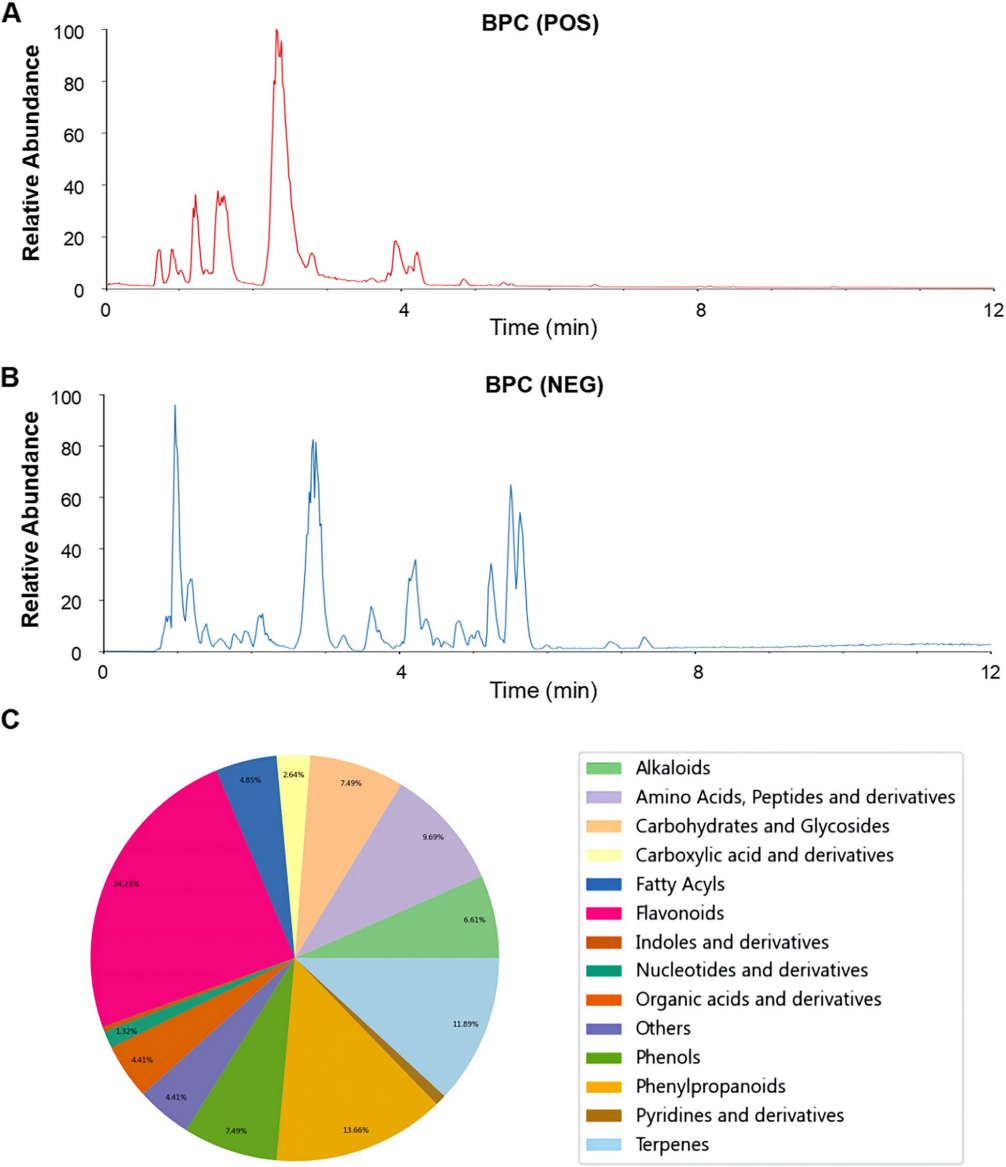
UPLC-MS analysis of ZSP-M. **A **Base peak chromatogram in the positive ion mode. **B **Base peak chromatogram in the negative ion mode. **C **Quantity distribution diagram of ZSP-M components

In order to clarify the chemical constituents of ZSP-M, UPLC-MS analysis was performed (Fig. 1A and B) and a composition quantity distribution diagram was constructed based on the data to provide a more visually comprehensive representation of the qualitative components (Fig. 1C). Flavonoids were the most abundant constituents, followed by phenylpropanoids terpenes, amino acids, peptides and derivatives. The main components of ZSP-M are listed in Table 1.

**Table 1:** The main components of ZSP-M characterized using UPLC-MS

NO	Metabolites	Formula	Ion mode	m/z	R.T (min)	Sort
1	Matrine	C15H24N2O	+	249.1959	2.31	Alkaloids
2	Oxymatrine	C15H24N2O2	+	265.1908	3.51	Alkaloids
3	Maackiain	C16H12O5	+	285.0752	8.15	Phenols
4	Oxysophoridine	C15H24N2O2	+	265.1908	3.94	Alkaloids
5	Scutellarin	C21H18O12	+	463.0861	4.84	Flavonoids
6	Chlorogenic acid	C16H18O9	-	707.1828	4.14	Phenylpropanoids
7	Caffeic acid	C9H8O4	+	163.0388	4.14	Organic acids and derivatives
8	Sucrose	C12H22O11	+	365.1051	0.9	Carbohydrates and Glycosides
9	Vitexin-2"-O-rhamnoside	C27H30O14	+	579.1697	4.7	Flavonoids
10	Miquelianin	C21H18O13	+	479.0812	4.8	Flavonoids
11	Trifolirhizin	C22H22O10	-	491.1197	5.93	Flavonoids
12	Rutin	C27H30O16	+	611.1597	4.71	Flavonoids
13	Chicoric acid	C22H18O12	-	947.1526	4.79	Phenylpropanoids
14	Puerarin apioside	C26H28O13	+	549.1594	4.33	Flavonoids
15	Puerarin	C21H20O9	+	417.1172	4.34	Flavonoids
16	Roseoside	C19H30O8	-	431.1925	4.48	Terpenes
17	Przewalskinic acid A	C18H14O8	+	359.0755	4.54	Phenylpropanoids
18	Hyperoside	C21H20O12	+	465.1024	4.82	Flavonoids
19	L-Proline	C5H9NO2	+	116.0708	0.92	Amino Acids and Peptides
20	Citric acid	C6H8O7	-	191.0199	0.96	Organic acids and derivatives
21	p-Synephrine	C9H13NO2	+	150.0913	1.14	Alkaloids
22	Pyroglutamic acid	C5H7NO3	+	130.0499	1.18	Amino Acids and Peptides
23	L-Tyrosine	C9H11NO3	+	182.0811	1.23	Amino Acids and Peptides
24	Adenosine	C10H13N5O4	+	268.1037	1.29	Nucleotides and derivatives
25	Succinic acid	C4H6O4	-	117.0196	1.37	Organic acids and derivatives
26	L-Leucine	C6H13NO2	+	132.1019	1.53	Amino Acids and Peptides
27	Gallic acid	C7H6O5	-	169.0145	1.79	Phenols
28	Neochlorogenic acid	C16H18O9	-	353.0879	3.72	Phenylpropanoids
29	Hydroxyphenyllactic acid	C9H10O4	-	181.051	3.81	Phenylpropanoids
30	Niga-ichigoside F1	C36H58O11	-	711.3964	5.57	Terpenes
31	Dihydroactinidiolide	C11H16O2	+	181.122	8.03	Terpenes
32	Salviaflaside	C24H26O13	-	521.1301	4.83	Carbohydrates and Glycosides
33	Vomifoliol	C13H20O3	+	207.1377	4.96	Others
34	Azelaic acid	C9H16O4	-	187.0978	5.39	Fatty Acyls
35	Ononin	C22H22O9	+	431.1327	5.54	Flavonoids
36	Salvianolic acid A	C26H22O10	-	493.1141	5.68	Phenylpropanoids
37	Sappanone A	C16H12O5	+	285.0752	5.92	Flavonoids
38	Soyasaponin Bb	C48H78O18	+	943.5243	8.33	Terpenes
39	Clinodiside A	C48H78O19	-	957.5069	6.32	Terpenes
40	Aurantiamide acetic acid	C27H28N2O4	+	467.1933	9.78	Amino Acids and Peptides

### Effect of ZSP-M on the 4NQO precancerous lesions

**Fig. 2:**
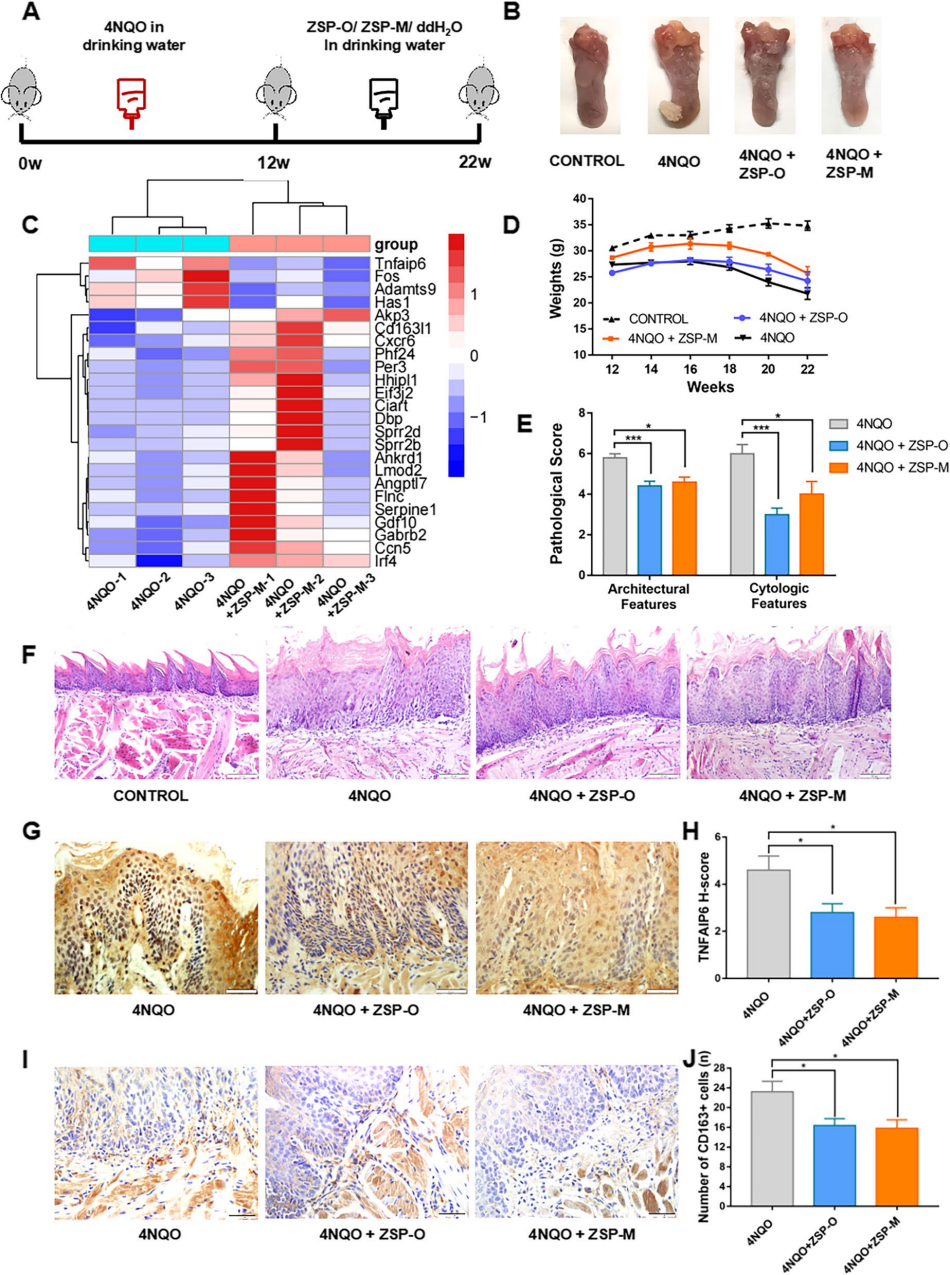
The effect of ZSP-M on 4NQO precancerous lesions. **A** Schematic diagram of the 4NQO precancerous lesion model in C57BL/6 mice. **B** Macroscopic view of tongue tissues. **C** Heat map of DEGs identified by RNA-seq with *P* < 0.01 in the 4NQO and 4NQO + ZSP-M groups. **D** Body weight at different time-points. **E** Pathological score of tongue tissues. **F** HE staining of tongue tissues (magnification 200 ×). **G**, **H **Immunohistochemical staining of TNFAIP6 protein expression (400 ×). **I**, **J **Immunohistochemical staining of CD163 protein expression (200 ×). Data represent the mean ± SE, * P < 0.05, *** *P* < 0.001

At the end of week 12 (4NQO intervention complete), the tongues of mice in all experimental groups generally appeared white and rough. Lesions similar to leukoplakia were observed, and no proliferative tumors were detected, indicating that the precancerous lesion model was successfully established. At the end of experimental week 22, the white plaque on the back of the tongue was more developed. Tongue masses of varying sizes were observed in most of the 4NQO group. In contrast, the texture of the white plaque was more uniform in the 4NQO + ZSP-M group (Fig. 2A and B). At week 16, weight loss in the 4NQO group was more rapid than that in the treatment groups (4NQO + ZSP-O/M), suggesting a better general condition in the treatment groups (Fig. 2D). HE-staining was performed to determine the degree of epithelial dysplasia. Both the architectural features and cytological features in the treatment groups were significantly less than those in the 4NQO group (*P* < 0.05) (Fig. 2E and F).

RNA-Seq was used to gain a global transcriptional view of the effect of ZSP-M treatment on the precancerous lesion. ZSP-M differentially regulated 71 genes (53 upregulated and 18 downregulated) compared with the 4NQO model group. A heat map of the DEGs with *P* < 0.01 is presented in Fig. 2C. The expression of the *Tnfaip6* gene exhibited the most significant decrease among them.

Compared to those in the 4NQO group, immunohistochemical staining revealed a decreased TNFAIP6 expression in the epithelium of mice in 4NQO + ZSP-O and 4NQO + ZSP-M groups (*P* < 0.05) (Fig. 2G and H). Additionally, there was a significant reduction in the number of CD163^+^ cells in both the epithelial and subepithelial tissues in the 4NQO + ZSP-O and 4NQO + ZSP-M groups (*P* < 0.05) (Fig. 2I and J).

### Effect on OSCC cells

**Fig. 3:**
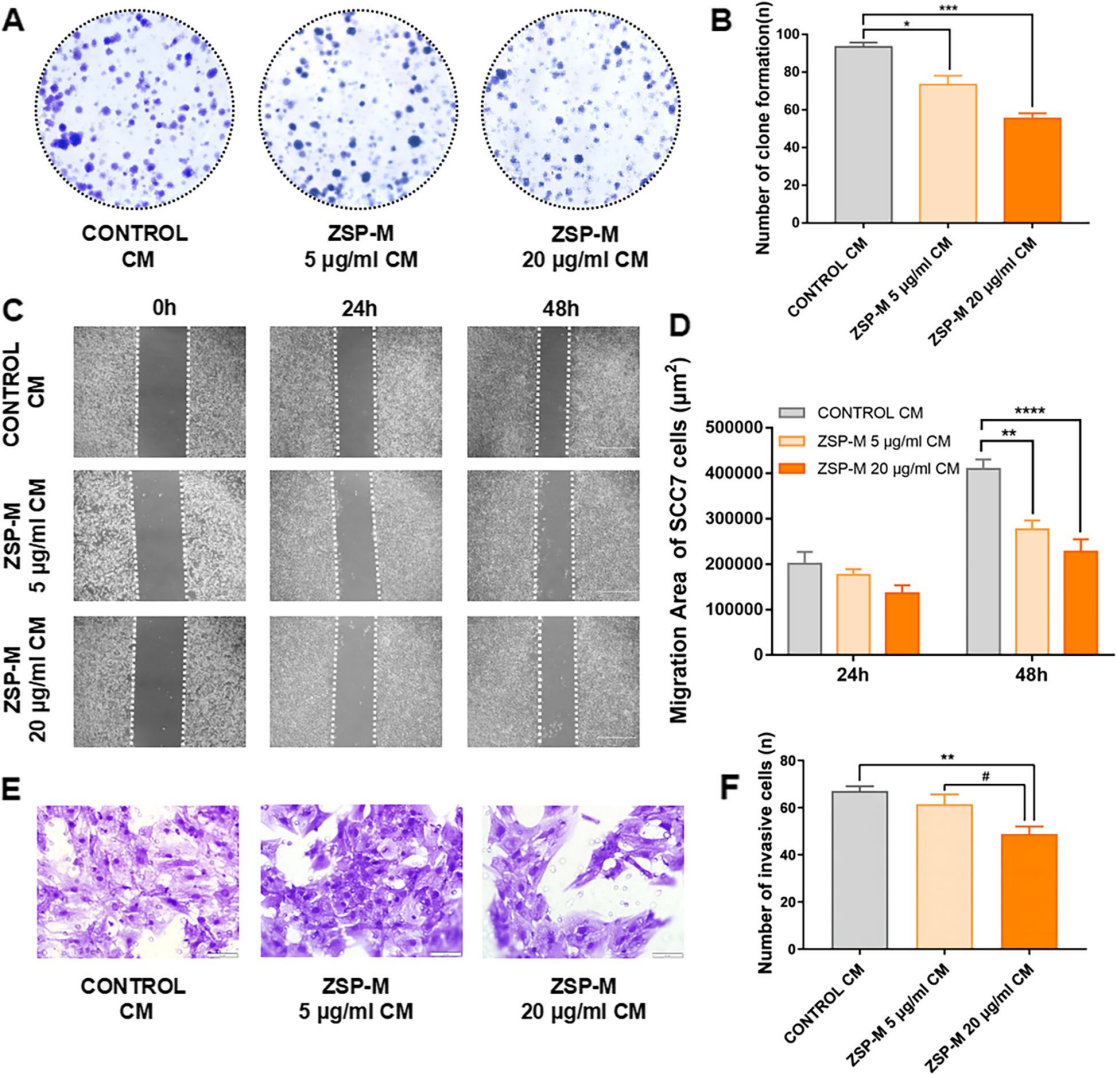
Effect of ZSP-M-induced CM on the biological properties of OSCC cells. **A**, **B **Colony formation assay results. **C**, **D **Scratch assay results. **E**, **F **Invasion assay results. Data represent the mean ± SE, *, ^#^* P* < 0.05, ** *P* < 0.01, *** *P* < 0.001, **** *P* < 0.0001

Colony formation assays demonstrated that the macrophage CM induced by 5 μg/mL and 20 μg/mL ZSP-M significantly decreased the number of SCC7 cell colonies, as compared to the normal macrophage CM (*P* < 0.05) (Fig. 3A and 3B).

Scratch assays demonstrated that SCC7 cell migration was significantly impeded by the macrophage CM induced by 5 μg/ml and 20 μg/ml ZSP-M at 48 h (*P* < 0.01). At 24 h, Although slight inhibition in the migration caused by ZSP-M was observed at 24 h, it did not reach the level of statistical significance (*P* > 0.05) (Fig. 3C and D).

Invasion assays demonstrated that the macrophage CM induced by 20 μg/ml ZSP-M significantly decreased the number of SCC7 cells that passed through the Transwell chamber compared to the normal macrophage CM (*P* < 0.01) (Fig. 3E and F).

### Effect on TAMs

**Fig. 4:**
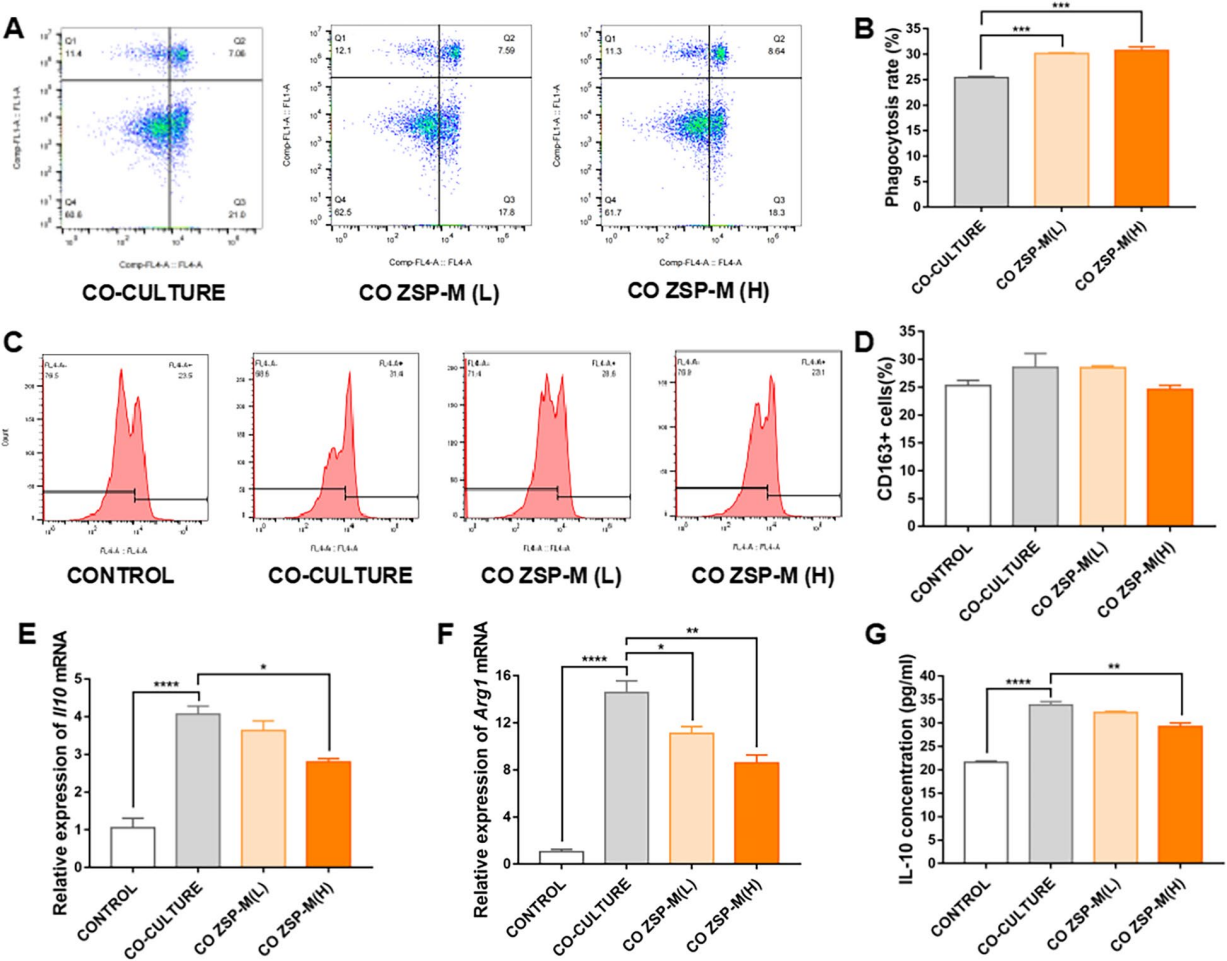
The effect of ZSP-M on TAMs within the co-culture environment. **A**, **B **Effect of ZSP-M on phagocytosis of OSCC cells by macrophages. **C**, **D **Effect of ZSP-M on the ratio of CD163.^+^ cells in TAMs in the co-culture environment. **E**, **F **Effect of ZSP-M on the relative expression of *Arg1* and *Il10* mRNA in TAMs. **G **Effect of ZSP-M on IL-10 cytokine in co-culture medium. Data represent the mean ± SE, * P < 0.05, ** *P* < 0.01, *** *P* < 0.001, **** *P* < 0.0001

Following pretreatment with ZSP-M at concentrations of 5 μg/ml and 20 μg/ml, phagocytosis assays demonstrated a significant increase in the proportion of RAW264.7 cells engaging in phagocytosis of SCC7 cells (*P* < 0.001) (Fig. 4A and B).

Under co-culture conditions, the proportion of CD163^+^ cells RAW264.7 cells was increased (*P* > 0.05), accompanied by a significant increase in the relative expression of *Il10* and *Arg1* mRNAs as well as IL-10 protein levels in the culture medium (*P* < 0.0001). ZSP-M mediated a slight inhibition of the rate of CD163^+^ cells at 5 μg/ml and 20 μg/ml (*P* > 0.05). It suppressed the relative expression of *Il10* and *Arg1* mRNAs in RAW264.7 cells, and also inhibited IL-10 protein levels of the culture medium. The inhibitory effect of ZSP-M was particularly pronounced at 20 μg/ml (*P* < 0.05). (Fig. 4C - G).

**Fig. 5:**
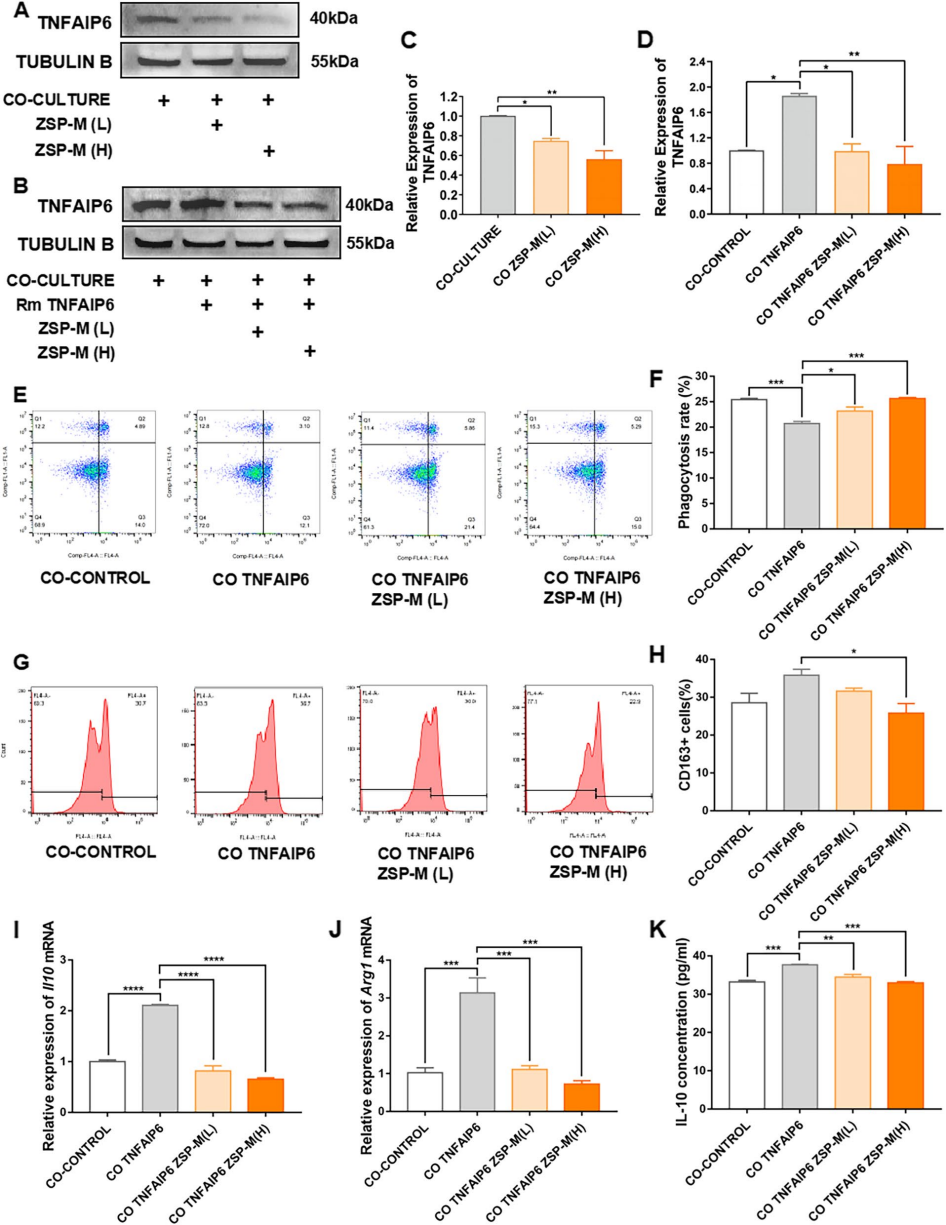
The ability of recombinant TNFAIP6 protein to rescue the effect of ZSP-M on TAMs within the co-culture environment. **A**, **B**, **C**, **D **Western blot analysis of the relative expression levels of TNFAIP6 protein. (Full-length blots are presented in Supplementary Fig. 1) **E**, **F **Effect of ZSP-M on phagocytosis of OSCC cells by macrophages. **G**, **H **Effect of ZSP-M on the ratio of CD163.^+^ cells in TAMs in the co-culture environment. **I**, **J **Effect of ZSP-M on the relative expression of *Arg1* and *Il10* mRNA in TAMs. **K **Effect of ZSP-M on IL-10 cytokine in the co-culture medium. Data represent the mean ± SE, * P < 0.05, ** *P* < 0.01, *** *P* < 0.001, **** *P* < 0.0001

Western blot analysis showed that ZSP-M reduced the relative expression of TNFAIP6 protein in RAW264.7 cells under co-culture conditions. After the addition of recombinant TNFAIP6 protein, there was a significant increase in the relative expression of TNFAIP6 protein in RAW264.7 cells. Meanwhile, ZSP-M still effectively suppressed the expression of TNFAIP6 protein. **(**Fig. 5A - D**)**.

The proportion of RAW264.7 cells engaged in phagocytosis of SCC7 cells exhibited a significant reduction following stimulation with recombinant TNFAIP6 protein, while a significant increase was induced by ZSP-M (*P* < 0.05) (Fig. 5E and F).

Induction by recombinant TNFAIP6 protein significantly enhanced the CD163 expression in RAW264.7 cells under co-culture conditions. ZSP-M inhibited the proportion of CD163^+^ cells, with a statistically significant difference observed at 20 μg/mL (*P* < 0.05). Recombinant TNFAIP6 protein significantly upregulated the relative expression of *Il10* and *Arg1* mRNAs in RAW264.7 cells within the co-culture environment (*P* < 0.001). In addition, there was a significant increase in IL-10 cytokine in the culture medium (*P* < 0.001). ZSP-M mediated an inhibition of the relative expression of *Il10* and *Arg1* mRNAs in RAW264.7 cells (*P* < 0.001), as well as IL-10 cytokine in the culture medium (*P* < 0.01). (Fig. 5G - K).

### Molecular docking and molecular dynamics simulation

**Fig. 6:**
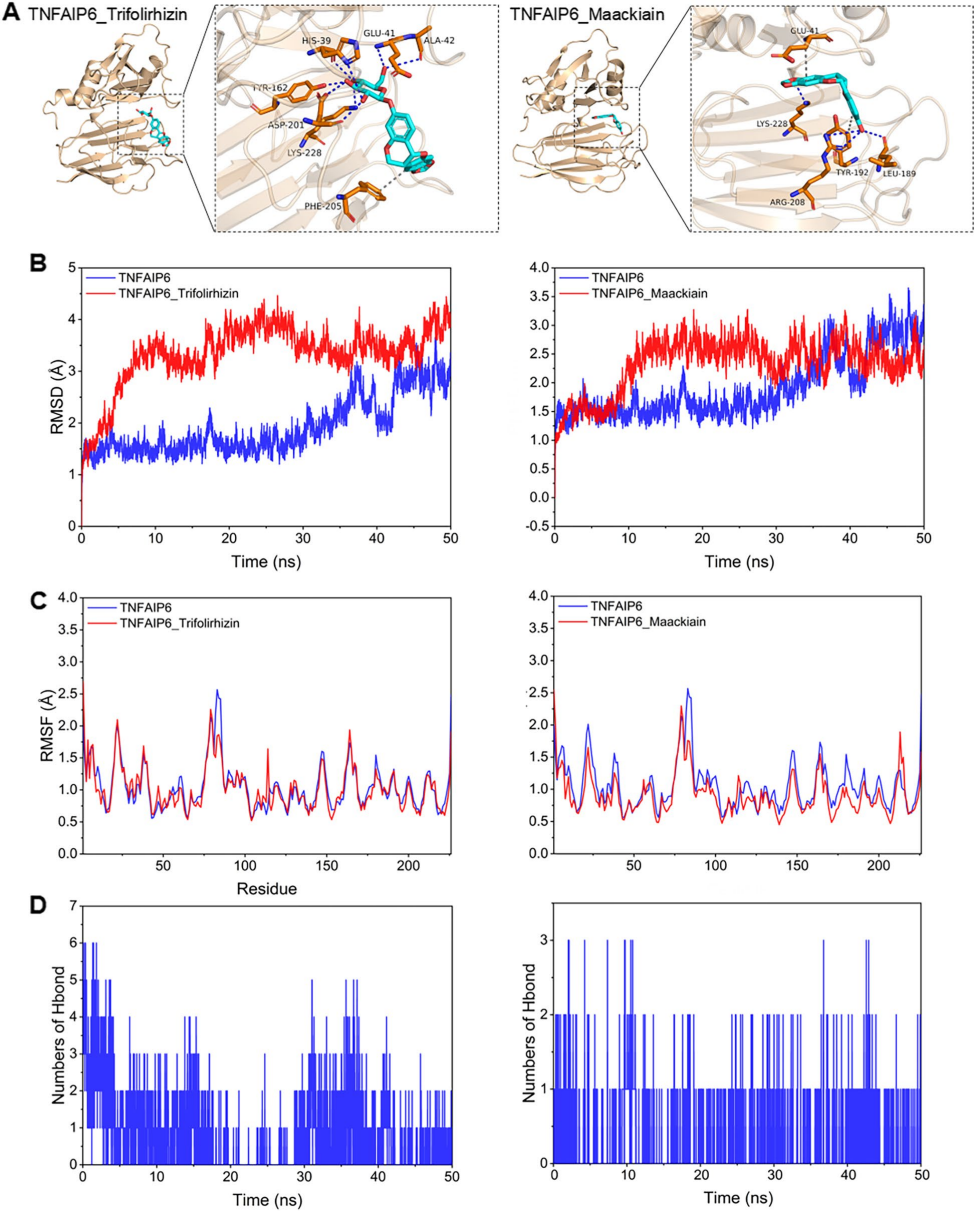
The molecular docking and molecular dynamics simulation outcomes of trifolirhizin and maackiain with TNFAIP6 protein. **A **Molecular docking mode diagram; blue dashed line represents hydrogen bonding and a gray dashed line indicates hydrophobic interaction. **B **RMSD of the complexes and TNFAIP6 protein. **C **RMSF of the complexes and TNFAIP6 protein. **D **Number of hydrogen bonds of the complexes and TNFAIP6 protein

The docking scores of molecular docking are presented in Supplementary Table 1. We selected two compounds that yielded significant outcomes to illustrate the findings of the computer simulations. Both trifolirhizin and maackiain bind the β-fold region of the TNFAIP6 protein. The interaction diagram shown in Fig. 6A revealed that trifolirhizin establishes hydrogen bonds with HIS-39, GLU-41, ALA-42, TYR-162, ASP-201, and LYS-228 of the protein, while engaging in hydrophobic interactions with PHE-205. Maackiain forms hydrogen bonds with ARG-208, LEU-189, and LYS-228 of the protein and participates in hydrophobic interactions with TYR-192 and GLU-41.

The docking scores of trifolirhizin and maackiain with TNFAIP6 were -8.3 kcal/mol and -7.6 kcal/mol, respectively, indicating the possibility of binding to TNFAIP6. Molecular dynamics simulation revealed that trifolirhizin binds TNFAIP6 with a binding energy of -16.85 ± 1.99 kcal/mol, driven primarily by electrostatic interactions, followed by van der Waals interactions and non-polar solvation free energy contributions. Similarly, maackiain interacts with TNFAIP6 with a binding energy of -14.31 ± 1.25 kcal/mol, mainly arising from van der Waals interactions, followed by electrostatic interactions and nonpolar solvation free energy (Table 2).

**Table 2:** Results of molecular docking and dynamics simulations (kcal/mol)

System name	TNFAIP6_Trifolirhizin	TNFAIP6_Maackiain
Docking score	-8.3	-7.6
ΔE_VDW_	-16.34 ± 3.95	-27.50 ± 4.41
ΔE_elec_	-60.67 ± 3.78	-5.89 ± 6.97
ΔG_GB_	63.95 ± 4.20	22.73 ± 3.44
ΔG_SA_	-3.76 ± 0.16	-3.62 ± 0.13
ΔG_bind_	-16.85 ± 1.99	-14.31 ± 1.25

ΔE_VDW_, ΔE_elec_, ΔG_GB_, ΔG_SA_ and ΔG_bind_ represent the Van der Waals interaction, electrostatic interaction, polar solvation free energy, nonpolar solvation free energy, and binding energy

The initial phase of the simulation revealed comparable RMSD fluctuations in the TNFAIP6_trifolirhizin complex, TNFAIP6_maackiain complex, and TNFAIP6 protein. In contrast, during later stages, it was evident that combining ligands resulted in significantly reduced RMSD fluctuations compared to when the ligands were not incorporated (Fig. 6B). The RMSF of the protein complexes was lower than when the ligands were not incorporated. These results demonstrated that trifolirhizin and maackiain stabilize the protein system, thereby enhancing activity (Fig. 6C). The number of hydrogen bonds in the TNFAIP6_trifolirhizin complex ranged from 0 to 6, surpassing that observed in the TNFAIP6_maackiain complex (0 to 3), suggesting that hydrogen bonding contributes significantly to the TNFAIP6_trifolirhizin complex formation (Fig. 6D).

## Discussion

Since the 1970s, ZSP-O has been applied for the prevention and treatment of gastrointestinal cancer with good results. It has also been found to exert a certain therapeutic effect on abnormal hyperplasia of the esophageal epithelium, intestinal metaplasia of chronic atrophic gastritis, and OPMD [28]. It also exhibits significant inhibitory effects on experimental tongue cancer, esophageal cancer, lung cancer, and medulloblastoma in mice [29, 30]. We have frequently used ZSP-O in the clinical treatment for OPMD, but had to discontinue its use due to obvious liver damage in several patients after long-term application. Although most clinical trials claimed that ZSP-O is safe, we consider that its widespread use has been hindered by the risk hepatotoxicity.

The previous study conducted by our research group identified diosbulbin B, obacunone, and fraxinellone as potentially hazardous components. These components were found to be present in the ethyl acetate phase of ZSP-O. Consequently, we obtained ZSP-M by eliminating the ethyl acetate phase [11, 12]. Preclinical investigations of ZSP-M involving pharmacodynamics and safety assessments were previously conducted in vitro and in hamster models [13, 14]. In this study, RNA-Seq analysis of mice tongue 4NQO precancerous lesion provided clear evidence that ZSP-M regulated immune-related genes and pathways while reducing the degree of abnormal hyperplasia. To understand the mechanism, we directed our attention toward *Tnfaip6*, a gene closely associated with the TNF pathway and exhibiting the most prominent downregulation. Also known as TNFα-induced protein 6, TNFAIP6 is a soluble protein belonging to the hyaluronic acid-binding protein family. It participates in extracellular matrix composition and remodeling, influences biological processes such as cell adhesion and migration, and plays a role in negative feedback regulation of inflammation [31]. TNFAIP6^+^ cells exhibit potent immunosuppressive activity by inducing the differentiation of macrophages from a pro-inflammatory phenotype to an anti-inflammatory phenotype [32]. Previous research has indicated that elevated TNFAIP6 expression may be linked to unfavorable prognosis in malignant tumors such as OSCC [33], gastric cancer [34], and glioma [35].

To demonstrate the efficacy of ZSP-M in targeting TNFAIP6, we performed molecular docking and dynamics simulations with the potential active ingredient and TNFAIP6, which confirmed their favorable interactions. Notably, maackiain has long been recognized as the principal bioactive compound of ZSP-O [36], while trifolirhizin and maackiain are interconvertible metabolites in vivo [37]. Our findings provide compelling evidence for the potential therapeutic utility of ZSP-M for targeting TNFAIP6. The lack of docking validation presents a limitation to this study, therefore, we plan to conduct a surface plasmon resonance experiment subsequently.

Macrophage infiltration is rare in normal oral mucosa epithelium, but is increased in OPMD like oral leukoplakia [38], and the immunosuppressive M2 phenotype macrophage infiltration (especially CD163^+^, but not CD204^+^ or CD206^+^) is associated with the deterioration of the disease [20, 39]. Macrophages recruited by tumors are regulated by their microenvironment and tend to exhibit the M2 phenotype, which may promote tumor angiogenesis, distant metastasis and continuous proliferation compared to the classically activated M1 phenotype macrophages [40, 41]. A meta-analysis of the correlation between head and neck squamous cell carcinoma and TAMs revealed a significant association between decreased CD163^+^ TAM infiltration and favorable overall survival [42]. These findings implicate CD163^+^ TAMs as a potential prognostic biomarker in OSCC. In our study, the expression levels of TNFAIP6 and CD163 in tongue precancerous lesions were downregulated following ZSP-M induction. Subsequent addition of recombinant TNFAIP6 protein in the co-culture environment resulted in upregulation of M2 macrophage markers, indicating a correlation between TNFAIP6 and M2 macrophages. In vitro experiments demonstrated that ZSP-M reduced TNFAIP6 expression and inhibited M2-type macrophage markers in TAMs. Based on these findings, we propose that the anticancer activity of ZSP-M is mediated through targeted inhibition of TNFAIP6 expression in TAMs, thereby modulating their phenotype and reducing the population of M2 macrophages.

## Conclusions

By employing a combination of UPLC-MS analysis, in vitro and in vivo experiments, as well as molecular simulation, the pharmacodynamic material basis of ZSP-M and the mechanism of its chemoprenentive effect on OSCC were elucidated in this study. We found that ZSP-M counteracts the immunosuppressive action of TAMs through its specific targeting of TNFAIP6, thereby exerting chemopreventive activity of OSCC. The current study provided experimental foundations for the clinical transformation of ZSP-M, and ZSP-M is expected to serve as a chemopreventive strategy for OSCC in the future.

### Supplementary Information

Supplementary Material 1.

Supplementary Material 2.

## Data Availability

The datasets analysed during the current study are available in the National Center for Biotechnology Information with the primary accession code PRJNA1134103.

## Abbreviations

OSCC: Oral Squamous Cell Carcinoma
ZSP-M: Modified Zeng Sheng Ping
TAMs: Tumor Associated Macrophages
UPLC-MS: Ultra-performance Liquid Chromatography-Mass Spectrometry
4NQO: 4-Nitroquinoline N-oxide
TNFAIP6: TNF Alpha Induced Protein 6
OPMD: Oral Potentially Malignant Disorder
TCM: Traditional Chinese Medicine
ZSP-O: Original Zeng Sheng Ping
DEGs: Differential Expressed Genes
ST: Sophora tonkinensis Gagnep
BO: Bistorta officinalis Delarbre
SA: Sonchus arvensis L
PV: Prunella vulgaris L
DB: Dioscorea bulbifera L
DD: Dictamnus dasycarpus Turcz
HE: Hematoxylin and Eosin
RNA-Seq: RNA Sequencing
CM: Conditioned Medium
RMSD: Root Mean Square Deviation
RMSF: Root Mean Square Fluctuation
SE: Standard Error
