# Supplementary figures and images for: Chemopreventive effect of modified zeng-sheng-ping on oral squamous cell carcinoma by regulating tumor associated macrophages through targeting tnf alpha induced protein 6
Source: BMC Complement Med Ther. 2024 Jul 27;24:287. doi: 10.1186/s12906-024-04593-0 (PMC11283705; doi:10.1186/s12906-024-04593-0)

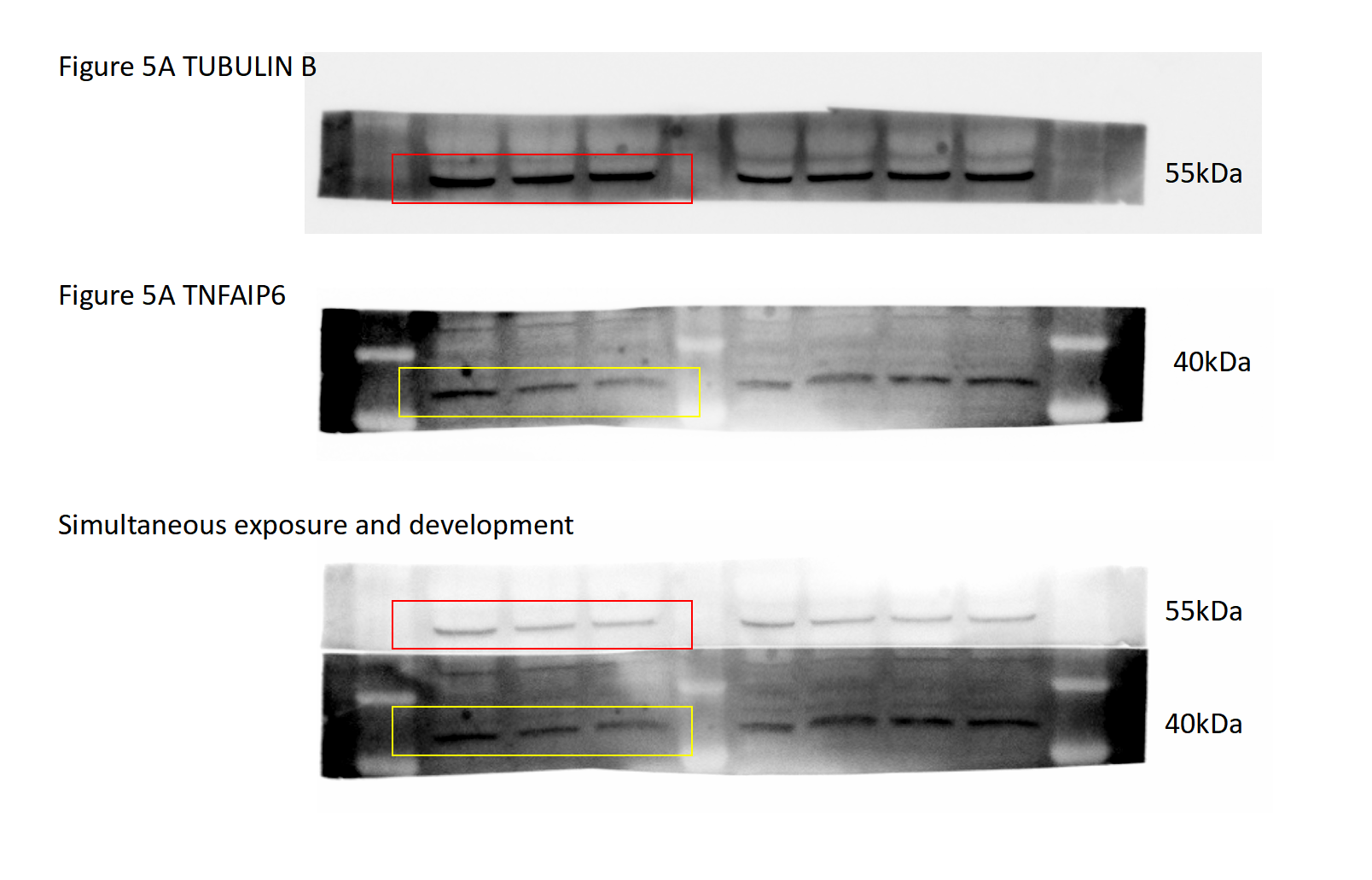


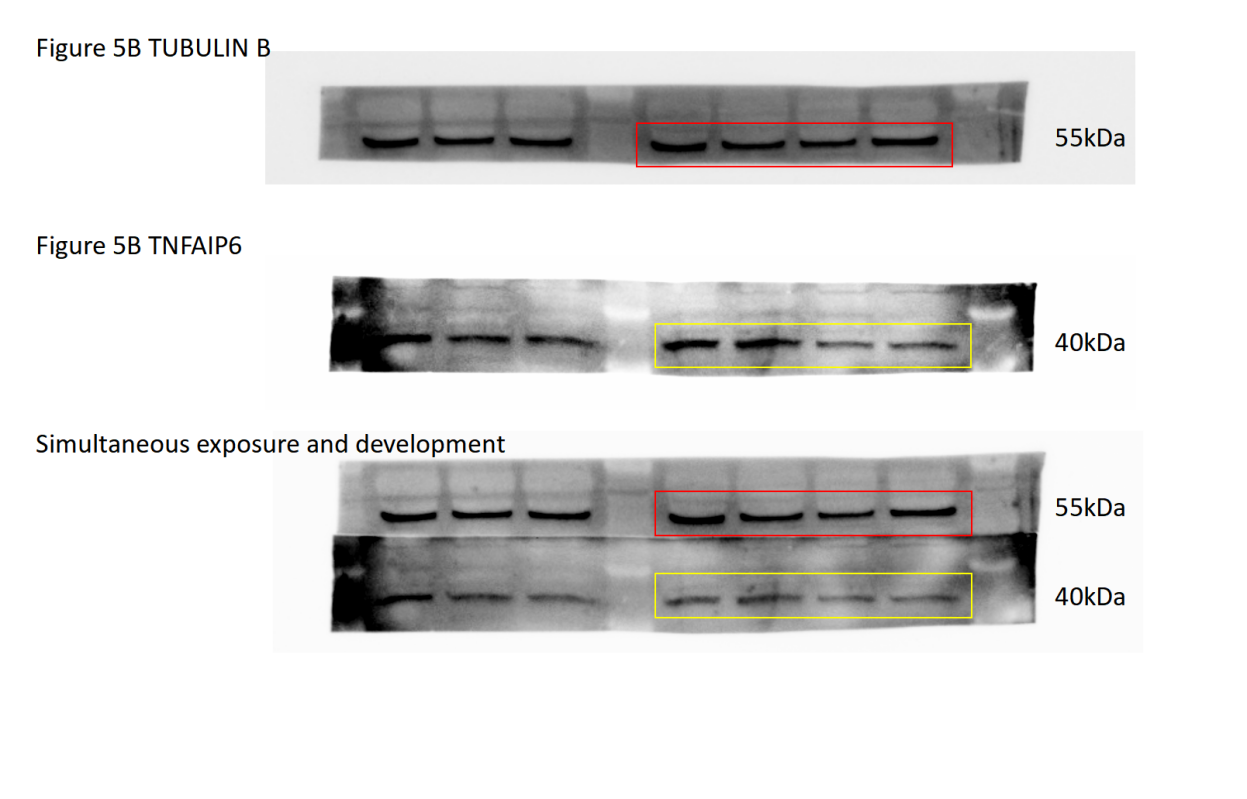

Supplement: Supplementary file 1 — Supplementary Material 1. [file 12906_2024_4593_MOESM1_ESM.docx]
