# Supplementary material for: Chemopreventive effect of modified zeng-sheng-ping on oral squamous cell carcinoma by regulating tumor associated macrophages through targeting tnf alpha induced protein 6
Source: BMC Complement Med Ther. 2024 Jul 27;24:287. doi: 10.1186/s12906-024-04593-0 (PMC11283705; doi:10.1186/s12906-024-04593-0)
Supplement: Supplementary file 2 — Supplementary Material 2. [file 12906_2024_4593_MOESM2_ESM.docx]

**Results of molecular docking (kcal/mol)**

| **NO.** | **Ligand** | **Docking score** |
| --- | --- | --- |
| 1 | Trifolirhizin | -8.3 |
| 2 | Maackiain | -7.6 |
| 3 | Matrine | -6.9 |
| 4 | Oxymatrine | -7.7 |
| 5 | Chlorogenic acid | -7.8 |
| 6 | Puerarin | -8.1 |
| 7 | Rutin | -8.1 |
| 8 | Chicoric-acid | -8.1 |
| 9 | Oxysophoridine | -8.3 |
| 10 | Miquelianin | -7 |
| 11 | Vitexin-2-O-rhamnoside | -6.8 |
| 12 | Caffeic acid | -5.9 |
